# Performance of simplified methods for quantification of [^18^F]NaF uptake in fibrodysplasia ossificans progressiva

**DOI:** 10.3389/fnume.2024.1406947

**Published:** 2024-07-22

**Authors:** Ruben Daniel de Ruiter, Esmée Botman, Bernd P. Teunissen, Adriaan Anthonius Lammertsma, Ronald Boellaard, Pieter G. Raijmakers, Lothar A. Schwarte, Jakko A. Nieuwenhuijzen, Dinko Gonzalez Trotter, Elisabeth Marelise W. Eekhoff, Maqsood Yaqub

**Affiliations:** ^1^ Department of Internal Medicine, Endocrinology Section, Amsterdam University Medical Centers, Vrije Universiteit Amsterdam, Amsterdam Movement Sciences, Amsterdam, Netherlands; ^2^ Department of Radiology and Nuclear Medicine, Amsterdam University Medical Centers, Vrije Universiteit Amsterdam, Amsterdam Movement Sciences, Amsterdam, Netherlands; ^3^ Department of Anesthesiology, Amsterdam University Medical Centers, Vrije Universiteit Amsterdam, Amsterdam, Netherlands; ^4^ Department of Urology, Amsterdam University Medical Centers, Vrije Universiteit Amsterdam, Amsterdam, Netherlands; ^5^ Regeneron Pharmaceuticals, Inc., Tarrytown, NY, United States

**Keywords:** fibrodysplasia ossificans progressiva, [^18^F]NaF, sodiumfluoride, PET, positron emission tomography, dynamic imaging

## Abstract

**Background:**

Fibrodysplasia Ossificans Progressiva (FOP) is a rare, genetic disease in which heterotopic bone is formed in muscles, tendons and ligaments throughout the body. Disease progression is variable over time and between individuals. ^18^F-fluoride uptake in newly formed bone can be evaluated using [^18^F]NaF (i.e., sodiumfluoride) PET/CT, identifying active areas of bone formation in FOP. The purpose of this study was to assess the performance of various semi-quantitative methods with full kinetic analysis.

**Results:**

Seven patients (age range: 20–31 years) with FOP underwent dynamic [^18^F]NaF scans at baseline and after one year. [^18^F]NaF uptake was measured in aorta descendens, vertebrae, heterotopic bone lesions and metabolically active regions on PET, and quantified using nonlinear regression (NLR) analysis together with standardized uptake value (SUV) and target-to-blood ratio (TBR). SUV was on measured the 40–45 min frame of the dynamic sequence (SUV^40–45^) and on the subsequent static sweep (SUV^Static^). Correlations between and SUV^40–45^ and NLR-derived *K_i_* were comparable when normalized to body weight (*r* = 0.81, 95% CI 0.64–0.90), lean body mass (*r* = 0.79, 95% CI 0.61–0.89) and body surface area (*r* = 0.84, 95% CI 0.70–0.92). Correlation between TBR^40–45^ and NLR-derived *K_i_* (*r* = 0.92, 95% CI 0.85–0.96) was higher than for SUV^40–45^. Correlation between TBR^40–45^ and NLR-derived *K_i_* was similar at baseline and after one year (*r* = 0.93 and 0.94). The change in TBR^40–45^ between baseline measurement and after one year correlated best with the change in NLR-derived *K_i_* in the PET-active lesions (*r* = 0.87).

**Conclusion:**

The present data supports the use of TBR for assessing fluoride uptake in PET-active lesions in FOP.

**Clinical trial registration:**

Sub-study of the Lumina-1 trial (clinicaltrials.gov, NCT03188666, registered 13-06-2017).

## Introduction

Fibrodysplasia ossificans progressiva (FOP) is a rare genetic disease affecting 0.88 per million individuals worldwide (1). Slowly but steadily, bone is formed in connective tissues such as muscles, ligaments and tendons throughout the body, leading to progressive immobility and ultimately an early death (2). In 2006, it was discovered that a single nucleotide substitution encoding the activin receptor type 1 (ACVR1)/activin receptor-like kinase 2 (ALK2) was the cause of FOP, and further genetic analyses revealed that nearly 97% of FOP cases are caused by the same mutation (c.617G>A, R206H), although other mutations have been described (3). Despite the fact that most patients share the same mutation, severity and rate of disease progression vary greatly and can be difficult to predict (4).

Heterotopic ossification in FOP is thought to take place in two separate manners. Classically, heterotopic bone in FOP is formed during so-called “flare-ups”, i.e., acute episodes of painful soft tissue swelling triggered by a minor trauma or an infection, but sometimes also occurring spontaneously (5). The initial local inflammatory response in muscle tissue is followed by endochondral ossification of the muscles and connective tissues (6). Recently, when analysing a series of MRI and PET/CT scans over time, areas were identified where heterotopic bone was formed without any typical flare-up symptoms, suggesting that a lower grade type of bone formation can also take place in patients with FOP, which is unrelated to the flare-ups (7). This was also confirmed in an ongoing natural history study in FOP, in which it was noted that nearly half of the patients reported instances of new heterotopic ossification (HO) without having experienced a flare-up (8). Treatment for FOP focuses on preventing soft tissue injury that can provoke a flare-up. Anti-inflammatory medication is often used empirically to reduce further bone formation once a flare-up has started, although there is little scientific evidence for this approach. Nevertheless, several therapeutic targets are being explored in multiple ongoing clinical trials, which will hopefully lead to better treatment and management of this devastating disease (9, 10).

Multiple tools have been developed to assess disease activity and progression of FOP. Assessment of joint mobility by goniometer measurements or cumulative scores such as the CAJIS (cumulative analogue joint involvement scale) reflect overall mobility and disease burden of a patient with FOP over time (11), but only moderately (*r* = 0.57) reflects the amount of heterotopic bone being formed (2). In clinical practice, however, it usually is more common to determine disease activity at the time of assessment for clinical decision making, rather than that it is based on changes over time. Clinical symptoms of a flare-up, such as pain, swelling, erythema and warmth, are non-specific, and it is difficult to predict whether the acute phase of the flare-up will result in HO formation or whether it will resolve itself (2). A multitude of potential biomarkers involved in inflammatory, chondrogenic and osteogenic processes have been investigated in patients with FOP, both during and in the absence of flare-up activity, and although some were markedly elevated in patients with FOP, none have shown to be capable of consistently reflecting disease activity and adequately predicting HO formation (12, 13).

Imaging techniques can be of great value in characterising disease activity and progression in FOP. Computed tomography (CT) scans can measure heterotopic bone volume over time, making it possible to evaluate disease progression. MRI (magnetic resonance imaging) and ultrasonography are able to detect soft tissue oedema associated with the inflammatory stage of HO, but they are less useful for detecting early bone formation and for accurately quantifying bone volume. Positron emission tomography (PET) is emerging as a new promising tool in FOP, as [^18^F]sodium fluoride [^18^F]NaF) PET can detect bone formation before it is visible on conventional CT (14).

Several methods exist to quantify [^18^F]NaF kinetics. A compartment model together with non-linear regression (NLR), as proposed by Hawkins et al., is considered to be the most accurate method for quantification of [^18^F]NaF uptake, but is also the most complex one (15, 16). The standardized uptake value (SUV) provides a simpler method towards assessing [^18^F]NaF uptake at any given time. SUV is the ratio of the image derived tissue radioactivity concentration divided by the whole body concentration based on the known dose of injected radioactivity, normalised to an anthropomorphic factor such body weight (BW). Though SUV is relatively easy to measure, it is prone to bias due to changes in blood flow and cannot assess the full range of [^18^F]NaF kinetics. Use of SUV to measure [^18^F]NaF uptake has already been validated against full kinetic NLR analysis in normotopic bone formation (17). Whether this simpler method can also be used to evaluate heterotopic bone formation and metabolism in FOP is unknown. Uptake in FOP may be markedly different from other metabolic diseases, given the genetic mutation interfering in the osteogenic pathways. In addition, drug therapies aimed at altering these pathways as part of a treatment for FOP may affect the rate of bone metabolism and, therefore, [^18^F]NaF kinetics even further. Potentially even more problematic, is that instead of altering bone metabolism, these drugs may also alter perfusion which, in turn, could give misleading results. For example, if metabolism does not change after therapy, but blood flow changes with 50%, SUV will also show an (erroneous) change of about 50%.

To assess various quantitative parameters reflecting [^18^F]NaF uptake in FOP, dynamic [^18^F]NaF PET/CT scans were performed in seven FOP patients who participated in the LUMINA-1 trial, evaluating [^18^F]NaF kinetics at baseline (T_0_) and after one year (T_1_) when all patients were receiving the trial drug garetosmab (an anti-activin A antibody).

## Material and methods

### Medical ethics

Ethical consent for this study was obtained from the Medical Ethics Review Committee of the VU University Medical Center. Patients were asked to participate in this study, and all patients provided written consent after being fully informed about the study purpose and any potential risks.

### Patient inclusion

Seven adults with the classic R206H FOP mutation underwent a dynamic [^18^F]NaF PET scan as part of the baseline measurements of the LUMINA-1 study. One year later, a second dynamic [^18^F]NaF PET scan was performed, at which point patients had received garetosmab for either 6 or 12 months.

### PET/CT data acquisition

All dynamic [^18^F]NaF PET scans were performed at the Amsterdam UMC, location VUmc. Scans were acquired using the Ingenuity PET/CT scanner (Philips Healthcare, Cleveland, OH, USA), which records planes over an axial field of view of 18.4 cm with voxels of 4 × 4 × 4 mm. First, a low dose CT scan was performed for attenuation and localisation purposes. Next, a bolus injection of 102 ± 3 (mean ± SD) MBq [^18^F]NaF was administered in the cephalic vein. Simultaneously, a 45 min dynamic (listmode) PET scan was started over the thoracic area, followed by a whole body scanning sweep (2 min per bed position, 10 bed positions). Venous blood samples were drawn at 10, 20, 30 and 45 min after injection. The dynamic acquisition was reconstructed in 36 consecutive time frames: 6 × 5, 6 × 10, 3 × 20, 5 × 30, 5 × 60, 8 × 150 and 3 × 300 s. The Philips Ingenuity system uses an iterative reconstruction algorithm, 3D-row action maximum likelihood algorithm (RAMLA), for the dynamic scans and rotationally symmetric volume elements ordered subsets time-of-flight (blob-os-tf) for the whole body scans (18). The combined CT and PET scans resulted in an estimated radiation dose of 4.7 mSv.

### Regional assessments

For the dynamic scans, the same volumes of interest (VOIs) were delineated at baseline and one year later by one reviewer (RDR). Independently, a second reviewer (BT, musculoskeletal radiologist) randomly segmented a third of the manually defined VOIs to assess inter-observer variability. Three VOIs of heterotopic bone were defined manually on the CT images, maintaining a cut-off of 80 Hounsfield units (HU) to distinguish between heterotopic bone and soft tissue, in line with previous studies (7). Based on an earlier PET/CT study on FOP, PET active lesions were identified using a SUV_peak_ (average SUV normalised for body weight within a 1 cm^3^ region of interest centred around the hottest voxel) >8.4 on the 40–45 frame at the end of the dynamic sequence, and then delineating three of these per scan using a semi-automatic tool. The semi-automatic tool included 50% of the SUV_peak_ in the designated area with adaptation for the local lesion to background contrast. As a reference for muscle tissue, one fixed-size VOI of 7.8 cm^3^ was defined in the triceps. As a reference for bone tissue 5 axial slices of the 8th thoracic vertebra were included, again maintaining a cut-off of 80 HU to distinguish between bone and soft tissue. An aorta VOI was manually delineated on 5 axial slices in the aorta descendens on the CT images for deriving an image derived input function and calculating target-to-blood ratios.

### Kinetic analysis

Analysis of [^18^F]NaF uptake was performed using the irreversible 2-tissue (2T3k) compartment model with 3 rate constants and an additional parameter for blood volume (*V_b_*), i.e., the preferred model for evaluation of [^18^F]NaF uptake in bone (17). Based on this model, the net influx rate constant *K_i_* was derived, reflecting the net rate of influx of [^18^F]NaF into bone. Image derived input functions were generated by projecting the aorta descendens VOIs on all frames. A multi-exponential function was then fitted based on the mean of the whole blood samples obtained at 20, 30 and 45 min. The plasma input function was determined assuming a fixed plasma-to-whole blood ratio of 1.21. The fixed value was based on an in-house unpublished data set for the same tracer from different earlier studies and is in line with plasma-to-whole blood ratios already published (19).

### Simplified measures

In addition to full kinetic analysis, ^18^F uptake was also analysed using simplified methods. SUV normalised for bodyweight (BW), body surface area (BSA) (20) and lean body mass (LBM) (21) were calculated using the final frame of the dynamic scan (40–45 min) and also using the static scan performed 45 min after injection with SUV_mean_ being the mean SUV of an VOI. Target to blood ratio (TBR) was calculated by dividing SUV_mean_ by SUV_mean_ of the aorta. Pearson correlation was used to compare the correlations of SUV_mean_ and TBR_mean_ with the gold standard, NLR-derived *Ki*, at baseline and after 1 year. The change in uptake between baseline and after 1 year measured by SUV_mean_ and TBR_mean_ was also correlated with the change measured by NLR-derived *Ki*.

### Statistical analysis

IBM SPSS statistics (IBM version 28) was used to perform statistical analyses. Correlations between various uptake values were tested through the Pearsońs correlation test. Inter-observer variability towards determining VOIs was evaluated through the intraclass correlation coefficient. GraphPad Prism 10 (GraphPad Software, San Diego, CA, USA) was used to perform linear interpolation. Solid lines in the graphs represent the mean interpolation lines with dotted lines representing the 95% confidence interval.

## Results

### Patient characteristics

After inclusion in the LUMINA-1 study, seven patients were scanned at baseline (T_0_) and after one year (T_1_) of trial participation. One scan could not be analysed, as no venous samples could be obtained through the venous catheter and, therefore, no calibration of the input function was possible. One scan failed, as a piece of heterotopic bone impinged on the vena basilica during the scan, obstructing the tracer from dispersing until the patient changed position after the scan. One scan could not be analysed due to movement of the patient during the scan, resulting in an unreliable image-derived input function. Thus, eleven scans were available for further analysis, six at baseline and five after one year of trial participation.

Ultimately, six patients (M/F: 2/4, age range: 20–31 years old) were included in these analyses. The CAJIS score (11) at baseline was mean (± SD) 11.3 ± 7.0 and after 1 year 11.3 ± 4.5, indicating that disease progression in all patients had already affected multiple joints (Table 1).

**Table 1:** Baseline characteristics and demographics study participants.

Patient characteristics	
Male, *n* (%)	4 (44%)
Age in years	
Mean, (SD)	25.4 (4.0)
Median (min–max)	26 (20–31)
CAJIS at baseline	
Mean (SD)	14.8 (7.0)
Median (min–max)	16 (6–26)

### Regional assessment

After manual segmentation of heterotopic bone on the CT images by one reviewer (RDR), a second reviewer (BT, musculoskeletal radiologist) manually segmented 12 of 36 (33.3%) randomly selected HO structures. A comparison of obtained volumes, TBRs and SUVs of the heterotopic bone lesions showed a near-perfect correlation between both observers (intraclass correlation coefficient = 0.99).

### Blood kinetics

**Figure 1:**
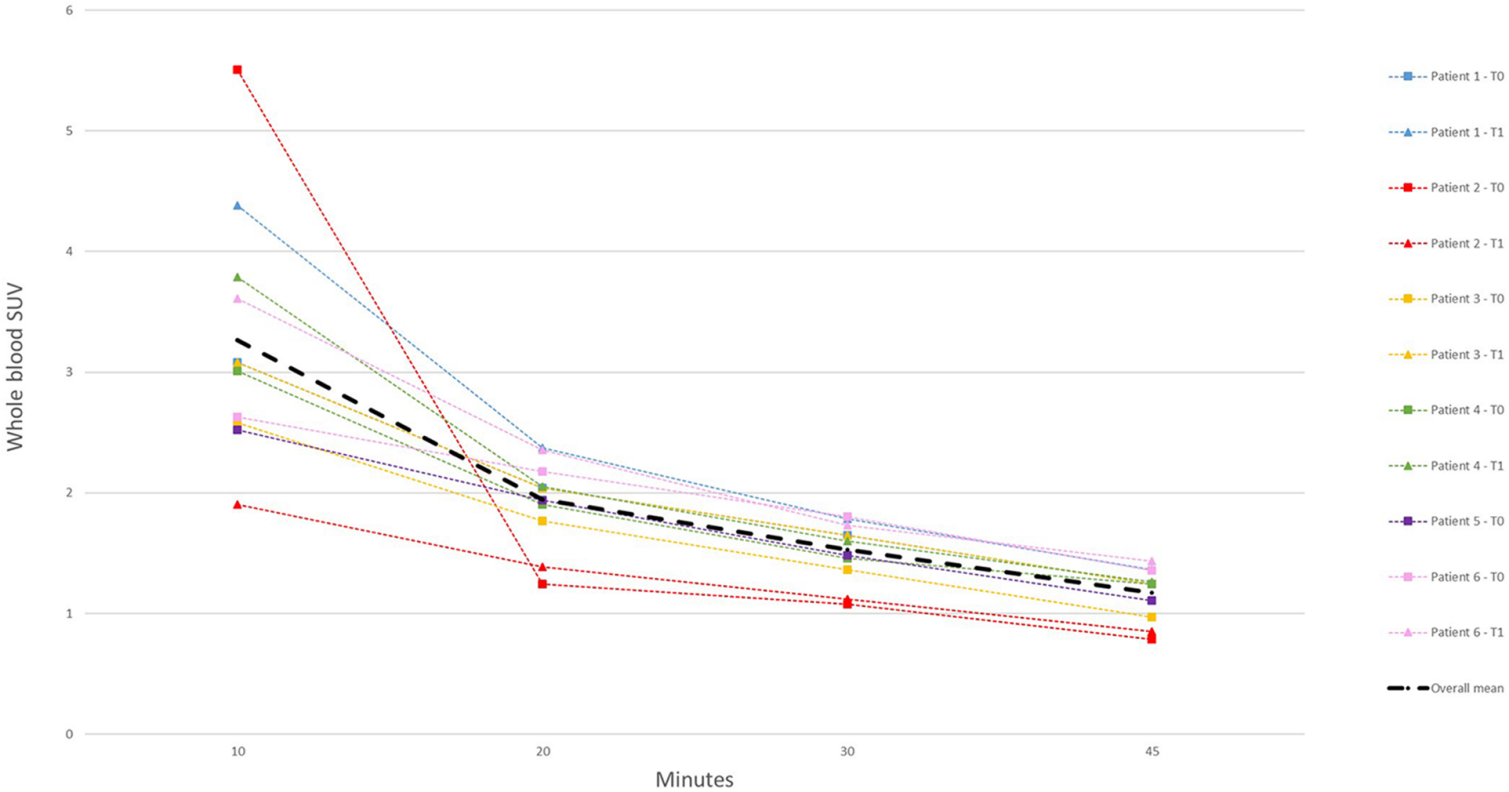
SUV of whole blood venous samples at various time points in all scans. The mean of all samples is represented by the thick line. T0, baseline; T1, after 1 year.

Radioactivity concentrations of venous blood samples are shown in Figure 1. These samples were used to calibrate the image-derived input function (IDIF). A multi-exponential function was fitted based on the mean of the whole blood samples with exclusion of the venous sample obtained at 10 min. The radioactivity concentration in the whole blood samples showed little intra-individual variability at baseline and after one year, indicating that garetosmab has little effect on tracer clearance.

### [^18^F]NaF uptake parameters

The correlation between SUV_mean_ and NLR-derived *K_i_* showed little dependency on the actual body normalisation factor (BW, LBM and BSA) being used, with Pearson's *r* values varying from 0.79 to 0.87 (Table 2).

**Table 2:** Scan intervals and correlation with NLR-derived K_i_ for all (semi)quantitative parameters at baseline.

Method	Interval (min)	Pearson's r correlation (95% CI)
NLR derived Ki	0–45	–
SUV mean BW	40–45	0.81 (0.64–0.90)
SUV mean LBM	40–45	0.79 (0.61–0.89)
SUV mean BSA	40–45	0.84 (0.70–0.92)
SUV mean BW	Static sweep	0.86 (0.72–0.93)
SUV mean LBM	Static sweep	0.87 (0.75–0.94)
SUV mean BSA	Static sweep	0.87 (0.81–0.93)
TBR mean	40–45	0.92 (0.85–0.96)
TBR mean	Static sweep	0.90 (0.82–0.93)

NLR, nonlinear regression; Ki, net rate of influx; BW, body weight; LBM, lean body mass; BSA, body surface area.

**Figure 2:**
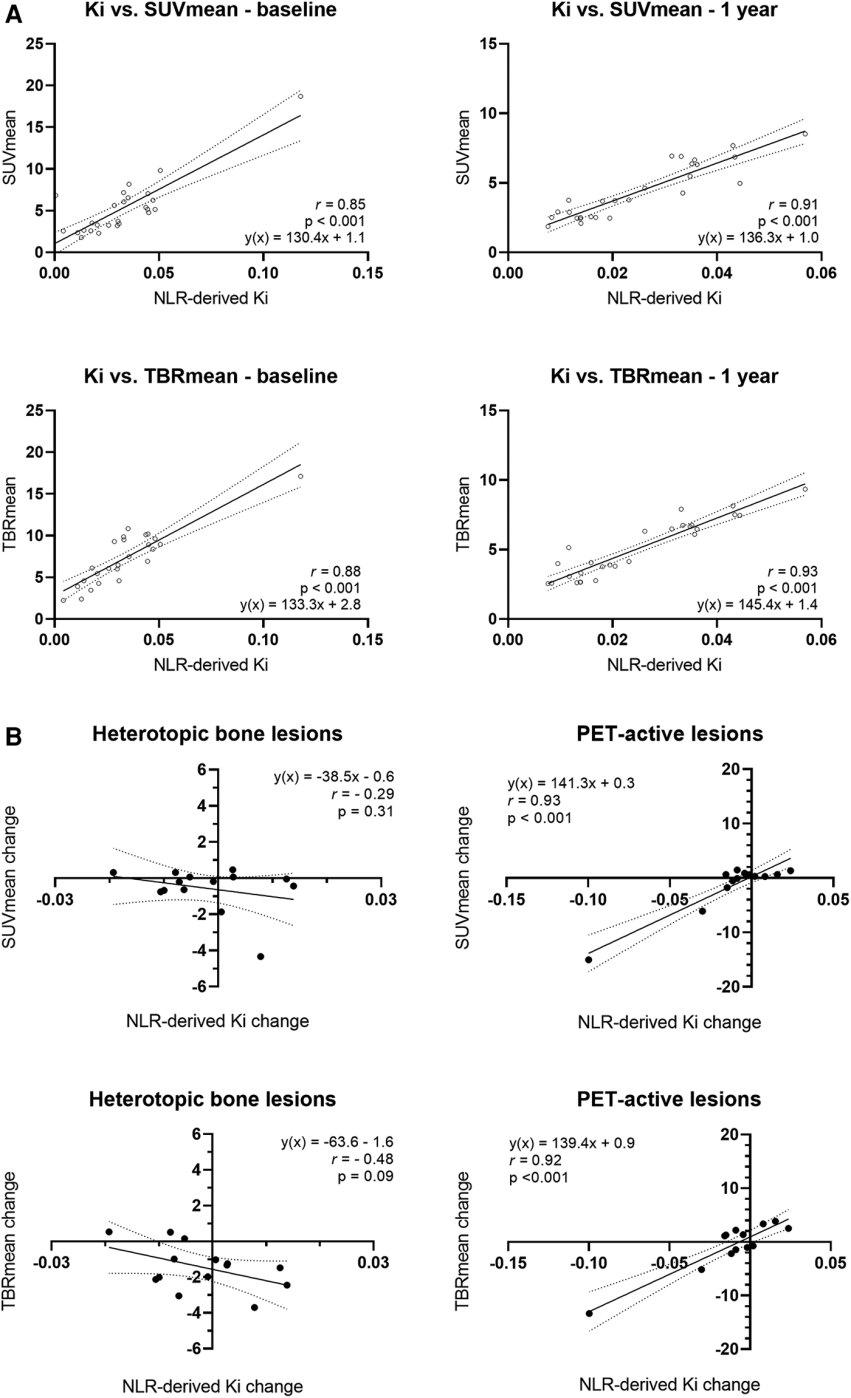
Relationship between F^18^ uptake as measured through NLR-derived K_i_ and the simplified parameters SUV_mean_ and TBR_mean_. (**A**) Correlation between NLR-derived K_i_ and simplified parameters derived from the 40–45 min frame (SUV_mean_^40–45^ and TBR_mean_^40–45^) at baseline and after one year in all areas of interest. (**B**) Correlation between the change in NLR-derived K_i_ at baseline and after 1 year and the change SUV_mean_ and TBR_mean_ at baseline and after 1 year. Subanalyses were performed for the heterotopic bone lesions and the PET-active lesions. The solid black line represents the mean agreement between the separate parameters, while the dotted black lines denote the boundaries of the agreement within a 95% confidence interval.

At baseline and after one year, SUVmean^40–45^ correlated with NLR-derived *Ki* with a Pearson's *r* of 0.85 and 0.95, respectively, and TBRmean^40–45^ correlated with a Pearson's *r* of 0.93 and 0.94 at baseline and after 1 year (Figure 2A). The change in SUVmean^40–45^ and TBRmean^40–45^ measured in heterotopic bone, correlated poorly with changes in NLR-derived *Ki*, with Pearson's of *r* = 0.42 and 0.57. In contrast, when examining PET-active regions, the change in SUVmean^40–45^ and TBRmean^40–45^ had a good correlation with NLR-derived *Ki* with Pearson's *r* of 0.82 and 0.91, respectively (Figure 2B). Correlations between SUVmean and TBRmean, derived from the static sweep with NLR-derived *Ki* were similar. At baseline and after one year, SUVmean^static^ correlated with NLR-derived *Ki* with a Pearson's *r* of 0.85 and 0.91, respectively, and TBRmean^static^ correlated with a Pearson's *r* of 0.88 and 0.93 at baseline and after 1 year (Supplementary Figure S1A). The change in SUVmean^static^ and TBRmean^static^ measured in heterotopic bone did not correlate with the change in the NLR-derived *Ki*. In contrast, when examining PET-active regions, the change in SUVmean^static^ and TBRmean^static^ had a good correlation with NLR-derived *Ki* with Pearson's *r* of 0.92 and 0.93, respectively (Supplementary Figure S1B).

## Discussion

Quantification of ^18^F-fluoride uptake may extend the scope of [^18^F]NaF PET/CT imaging in FOP from early detection of areas of increased heterotopic bone formation to monitoring disease activity and response to therapeutic interventions. Simplified scanning protocols, without the need for arterial or venous sampling could make it feasible to use [^18^F]NaF PET/CT scanning for routine assessment of FOP patients. In a cross-sectional analysis, both SUV_mean_ and TBR_mean_ showed a strong correlation with NLR-derived K_i_, both before and during treatment with garetosmab. When evaluating the same lesions over time, changes in fluoride uptake as measured by TBR_mean_ best reflected the change in fluoride uptake as measured by NLR-derived *Ki*.

To the best of our knowledge, this is the first study performing dynamic [^18^F]NaF scans in patients with FOP. Due to the nature of the disease, there are a few considerations that need to be mentioned. Obtaining an image-derived input function through continuous arterial sampling, the preferred option for dynamic scanning, has an unacceptable high risk of causing heterotopic ossification in FOP. We therefore opted for venous sampling, but were restricted to 1 ml per sample, to do as little harm as possible. In 1 ml of whole blood it is possible to accurately measure the [^18^F]NaF concentration in MBq/ml, though for conversion to plasma levels a fixed value of 1.21 was assumed based on an in-house unpublished data set for the same tracer from different earlier studies, though other studies have found similar plasma-to whole blood ratios (19). Secondly, most PET/CT scanners currently in use have axial fields of view ranging from 15 to 30 cm, limiting the body area that can be scanned at any given time. By moving the scanner bed during the scan, tracer uptake throughout the body can be visualized, providing a so-called (static) whole body PET/CT scan. In dynamic PET/CT scans, the position of the scanner bed is fixed, as tracer uptake needs to be monitored continuously over the same position. As a consequence, the field of view available for assessment of lesions in dynamic PET/CT scans is limited. To investigate the rest of the body, often a whole static sweep is performed after the dynamic sequence, making it a necessity to assess the performance of simplified parameters such as SUV and TBR. Fortunately, new total body PET/CT scanners have been developed, expanding the field of view to one to two meters. This is of particular interest for conditions where lesions are distributed throughout the body, such as in FOP, and this generation of scanners should therefore be considered for use in future studies when possible.

Despite the limited field of view in our study, we were still able to identify multiple active PET lesions and heterotopic bone in all participants, making it possible to evaluate the correlation between [^18^F]NaF kinetics and various semi-quantitative parameters. FOP is a heterogeneous disease spread throughout the musculature of the body and which progresses at an unpredictable pace (8). A piece of heterotopic bone may be growing in one location, whilst remaining stable elsewhere. In the present study, various volumes of interest were defined, i.e., CT-based VOIs, in which heterotopic bone was delineated, and PET-based VOIs in which active lesions were defined based on [^18^F]NaF uptake. From a clinical perspective, it makes more sense to follow the PET-active lesions rather than existing stable heterotopic bone with regard to disease activity. These are the active areas that are not bone yet, but may rapidly be so. Moreover, the changes in TBR_mean_ and SUV_mean_ better reflected the changes in the net rate of [^18^F]NaF uptake, in these PET-active lesions. We recommend future studies to focus on measuring uptake in PET active regions and monitor them over time by measuring TBR. Following (systemic) therapy, the correlation between semi-quantitative measures and true [^18^F]NaF kinetics should be re-evaluated, as therapy may affect the underlying biology (e.g., perfusion) in which case simple baseline and follow-up uptake measurements may not be comparable with each other.

The better performance of TBR is likely due to variation in the blood background activity, which is accounted for in the TBR calculation, but not in the SUV calculation. TBR is a semi-quantitative parameter frequently used in PET studies of vascular inflammation and atherosclerotic plaques. Blood activity of the tracer can vary between patients due to biological factors affecting its distribution, uptake and excretion (22). Uptake of an irreversible tracer is directly proportional to perfusion, so both SUV and TBR will be affected by changes in perfusion. Locally, vascularization varies throughout the endochondral ossification process. Initially, a hypoxic poorly vascularized area is present during the cartilaginous phase. This hypoxia, in turn, promotes neovascularization of the cartilage as it transitions to the ossifying phase (6, 23, 24). The normalization of [^18^F]NaF uptake to blood activity, being part of TBR, corrects for any variation in [^18^F]NaF blood supply towards the target tissue.

In a few cases, [^18^F]NaF PET/CT has already been used to evaluate ossifying activity in FOP. Elevated SUV was reported in a FOP patient after jaw surgery, preceding subsequent heterotopic ossification (14). Later, a series of 13 [^18^F]NaF PET/CT scans in 5 different patients were examined, and areas with increased ^18^F-fluoride uptake were identified, which subsequently corresponded with radiological progression of HO on a later scan (7). Areas with increased fluoride uptake, measured by SUV, were found to be associated with an increase in heterotopic ossification as measured by CT. A SUV_peak_ higher than 8.4 was found to be indicative of new HO on a later scan, though the increment of the SUV did not appear to correlate with the amount of bone volume formed. The higher correlation of TBR with full kinetic analysis in the present study, suggests that TBR may be better suited as a semi-quantitative parameter for predicting heterotopic bone formation. Ideally, longitudinal studies with multiple [^18^F]NaF PET/CT scans need to be performed to determine the optimum cut-off of TBR with regard to sensitivity and specificity for predicting heterotopic ossification in FOP. Most trials in FOP have used the change in total HO volume, as assessed by low-dose whole body CT, as the primary outcome measure for reflecting disease activity and thus drug effectiveness. In addition to the measured HO volume on CT, PET active lesions can be followed over time, visualizing and quantifying disease activity before they are seen on CT images. Other outcome measures reflecting disease activity in FOP include the number of new HO lesions as assessed by CT, number of body regions with HO, flare-up incidence, quality of life questionnaires and clinical measurements, such as CAJIS and pulmonary function tests (NCT03312634, NCT02190747, NCT03188666, NCT04307953). Two trials have also included the number of active PET lesions and lesion activity as outcome measures (NCT03188666, NCT04307953).

## Conclusions

Our present study supports TBR as the most suitable semi-quantitative parameter to measure change in ^18^F-fluoride uptake in patients with FOP, given the high correlation with NLR-derived *K_i_* and the short scanning time required. In addition, changes in uptake are best evaluated in small PET active regions rather than over larger areas of heterotopic bone. Separate studies with dynamic scanning should be performed if there is reason to believe that ^18^F-fluoride kinetics may be altered by the therapy itself, rather than as a result of a change in bone metabolism reflecting disease activity.

## Data Availability

Qualified researchers may request access to study documents that support the methods and findings reported in this manuscript. Individual anonymized patient data will be considered for sharing once the product and indication has been approved by major health authorities (for example, US FDA, European Medicines Agency and the Pharmaceuticals and Medical Devices Agency), if there is legal authority to share the data and there is not a reasonable likelihood of patient re-identification. Requests should be submitted to https://vivli.org/ (the typical response time is 6-12 months).
