# Supplementary material for: Performance of simplified methods for quantification of [18F]NaF uptake in fibrodysplasia ossificans progressiva
Source: Front Nucl Med. 2024 Jul 22;4:1406947. doi: 10.3389/fnume.2024.1406947 (PMC11460293; doi:10.3389/fnume.2024.1406947)
Supplement: Supplementary file 1 [file Image1.pdf]

Supplementary Material

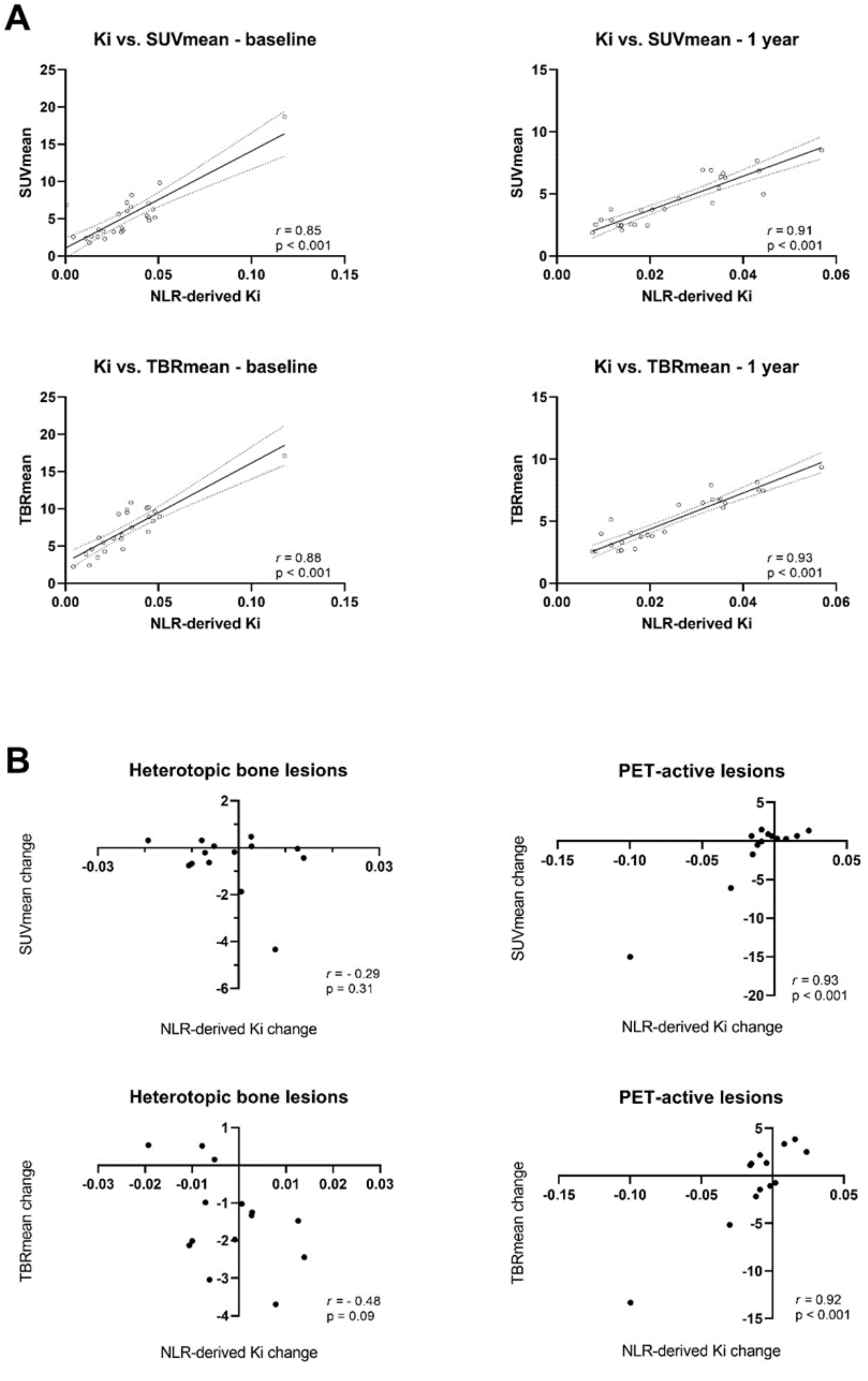

Supplementary Figure 1 –

Relationship between F^{18} uptake as measured through NLR-derived K_i and the simplified parameters SUV_{mean} and TBR_{mean}. (A) Correlation between NLR-derived K_i and simplified parameters derived from the

static sweep at baseline and after one year in all areas of interest. (B) Correlation between the change in NLR-derived K_i at baseline and after 1 year and the change SUV_{mean} and TBR_{mean} at baseline and after 1 year. Subanalyses were performed for the heterotopic bone lesions and the PET-active lesions. The solid black line represents the mean agreement between the separate parameters, while the dotted black lines denote the boundaries of the agreement within a 95% confidence interval.
